# Radiologic patterns of distant organ metastasis in advanced breast cancer patients: Prospective review of computed tomography images

**DOI:** 10.1002/cnr2.1988

**Published:** 2024-02-13

**Authors:** Bashiru Babatunde Jimah, Emmanuella Amoako, Emmanuel Owusu Ofori, Patrick Kafui Akakpo, Luke Adagrah Aniakwo, Kofi Ulzen‐Appiah, Emmanuel Gustav Imbeah, Martin Tangnaa Morna, Patience Koggoh, Harry Akligoh, Randy Tackie, Aida Manu, Lily Paemka, Benjamin Dabo Sarkodie, Asare Kweku Offei, David Hutchful, Joyce Ngoi, Yaw Bediako, Ganiyu Adebisi Rahman

**Affiliations:** ^1^ Department of Medical Imaging University of Cape Coast Cape Coast Ghana; ^2^ Yemaachi Biotech Accra Ghana; ^3^ Department of Surgery Cape Coast Teaching Hospital Cape Coast Ghana; ^4^ Department of Pathology University of Cape Coast Cape Coast Ghana; ^5^ Department of Pathology Cape Coast Teaching Hospital Cape Coast Ghana; ^6^ ACT Pathology Services Cape Coast Ghana; ^7^ Department of Surgery University of Cape Coast Cape Coast Ghana; ^8^ Department of Radiology, School of Medicine and Dentistry University of Ghana Accra Ghana; ^9^ Department of Surgery Korle Bu Teaching Hospital Accra Ghana

**Keywords:** abdomen and pelvis, breast cancer, computer tomography, liver metastasis, metastasis, radiology

## Abstract

**Background:**

Breast cancer (BC) metastases to the abdomen and pelvis affect the liver, mesentery, retroperitoneum, peritoneum, bladder, kidney, ovary, and uterus. The study documented the radiological pattern and features of the chest, bone, abdominal and pelvic (AP) metastases among advanced BC patients.

**Aim:**

The aim is to document the radiological pattern and features of breast cancer metastasis in the chest, abdomen, pelvis and bones.

**Materials and Results:**

Chest, abdominal, and pelvic computed tomography scan images of 36 patients with advanced BC were collated from Cape Coast Teaching Hospital and RAAJ Diagnostics. The images were prospectively assessed for metastasis to the organs of the chest, AP soft tissues, and bones. Radiologic features of metastasis of the lungs, liver, lymph nodes (LNs), and bones were documented. Patients' demographics, clinical data, and histopathology reports were also collected. The data were captured using UVOSYO and exported to Microsoft Excel templates. The data obtained were descriptively analyzed. Only 2.8% of BCs exhibited metaplastic BC, whereas 97.2% had invasive ductal BC. Triple‐negative cases were 55.6%. Of 36 patients, 31 (86.1%), 21 (58.3%), and 14(38.8%) were diagnosed of chest, AP, and bone tissues metastasis, respectively. LN involvement was reported in 26 (72.2%) patients. Majority, 21 (58.3%) were diagnosed of multiple sites metastasis with 15 (41.7%) showing single site. Lungs (77.4%, 24/31) and liver (47.6%, 10/21) were the most affected distant organs. Most bone metastases were lytic lesions (92.9%, 13/14) with the vertebrae (85.7%, 12/14) been the most affected.

**Conclusion:**

According to the study, advanced BC patients have a higher‐than‐average radiologic incidence of lung, liver, bone, and LN metastases.

## 1 INTRODUCTION

Breast cancer (BC) is the most common cancer and the leading cause of cancer‐related deaths in women worldwide.^1^, ^2^, ^3^ The incidence of BC has been increasing progressively over the last few decades.^2^, ^4^, ^5^

Most often, distant metastases of BC are not observed during the initial diagnosis. Less than 10% of all newly diagnosed BC patients will have obvious distant metastatic disease at the time of initial diagnosis.^6^ BC metastases may arise between 2 and 5 years after the initial diagnosis if not well managed.^2^, ^7^, ^8^ The most frequently associated sites of distant tumor metastasis are the liver, lung, bone, soft tissue, and adrenal glands.^7^, ^9^, ^10^, ^11^

Imaging modalities such as ultrasound and computed tomography (CT) are suitable screening and diagnostic tools for monitoring cancer diseases.^6^, ^12^, ^13^ Radiological assessment of BC metastasis is crucial for the successful staging and management of patients. CT is a common modality used to evaluate the chest, abdominal viscera, pelvic soft tissues, and related bones.^6^, ^13^ In practice, contrast medium is utilized to enhance the radiologic features of pathologies observed in noncontrast procedures. The literature shows the varying imaging features of BC metastases, even though they may conform to certain patterns in some organs.^7^

Aggressive BC is well documented in women of the Black African race, including those from Ghana.^14^, ^15^, ^16^, ^17^ Routine radiologic examinations for metastases should be encouraged to promote early detection of cancer spread to local and distant sites for management. This is often applicable to CT scans for patients with untreated advanced BC, which is different from CT scans for all BC patients, including those with early‐stage disease, and for follow‐up purposes. There is limited relevant literature to better understand lungs, hepatic, kidney, intra‐abdominal, and pelvic metastatic patterns in BCs in developing countries. Literature on metastatic BC is frequently reported from developed countries where technological advancement is evidently better.^18^, ^19^, ^20^, ^21^, ^22^ In this study, the radiological pattern and features of the chest, bone, abdominal and pelvic (AP), and lymph node (LN) metastases among patients with advanced BC were examined, which is frequently reported in developed countries where technological advancement is evidently better.

## 2 METHODS

### 2.1 Study design

This was a cross‐sectional study of patients with advanced BC who were recruited with informed consent for the AMBER01 Breast Cancer Project at the RAAJ Diagnostics and Cape Coast Teaching Hospital between January 2021 and December 2022.

### 2.2 Study subjects

The study included 36 treatment‐naive Ghanaian women with metastatic BC (Stage IIIB and IV) at the Cape Coast Teaching Hospital. Twenty‐three patients resided in Central region (same region CT facility is situated) whereas 14 patients resided out the Central region (outside the region CT facility is situated). However, only eight reside in Cape Coast, the same city CT facility is located. All cost including CT scan and transportation to and from the CT scan center were sponsored by the investigators, the participants did not bear any cost. These patients were initially recruited for the AMBER01 liquid biopsy study to detect actionable mutations. All individuals suspected of having a breast lump undergo a series of examinations such as biochemistry tests and a histological examination. The results of these tests included the metastatic tumor's type and presence, as well as the grade and biochemical markers of the original tumors. This study, however, was limited to patients who were referred to our specialized diagnostic center in Cape Coast, Central Region, for CT imaging and had advanced metastatic breast tumors. The CT scan was performed to identify distant organs implicated by the metastatic cancer.

### 2.3 Imaging diagnostic criteria for positive metastasis

We developed imaging diagnostic criteria for positive metastasis cases based on imaging findings of previous studies.^20^, ^23^, ^24^ Patients met two criteria to be eligible: (i) they had surgical biopsy or metastatic site surgery resulting in histological confirmation of BC, and (ii) they were referred to our diagnostic centers for imaging tests to confirm the presence of metastases to the chest and abdominopelvic regions during the initial staging or follow‐up of BC.

*Lung metastasis CT diagnosis criteria*: Lung lesions were considered as lung metastases when at least one of the following criteria was present: (i) CT showed typical imaging features such as solitary or multiple nodules of variable size, (ii) endobronchial lesion, (iii) air space consolidation, (iv) focal lung opacities with a history of breast cancer, (v) lymphangitic carcinomatosis, and (vi) ipsilateral pleural effusion of BC laterality.
*Liver metastasis CT diagnosis criteria*: A patient is noted to have liver metastasis if hepatic CT imaging showed features such as (i) solitary or multiple nodules of variable size, (ii) irregular hypodense nodules of peripheral enhancement, and (iii) tumor infiltrates and spreads along the hepatic sinusoids.
*Bone metastasis CT diagnosis criteria*: CT imaging showed features of bone tissue degeneration either sclerotic or lytic.
*LNs metastasis CT diagnosis criteria*: LNs were considered as metastatic when at least one of the following criteria was present: (i) round or oval LN, (ii) enhancement of the LN, and (iii) loss of the fatty hilum.

### 2.4 Data collection

A total of 32 patients underwent CT of the abdomen, pelvis, and chest, and 4 patients underwent US scan examinations of the abdomen and pelvic regions to assess BC metastasis. Only one CT scan procedure was done per participant. A radiology specialist with 7 years of experience in CT imaging performed the CT scans and reports. Images of all 36 patients were assessed and analyzed. Three impartial assessors—two physicians and one radiologist—reviewed the CT reports and related images using a single‐blinded method. Participants were unaware of who was examining what, however these evaluators worked together and reached consensus on CT results. The images were prospectively assessed for BC metastasis to the lungs, liver, kidney, spleen, peritoneum, uterus, and ovaries. Metastatic LN involvement has also been reported. Other variables, such as patient demographics (e.g., age) and clinicopathological features of the primary breast tumors were collected. Data were captured using UVOSYO, a clinical data collection tool.

### 2.5 Data analysis

The data were exported to the Statistical Package for Social Sciences (IBM SPSS version 24) and descriptively analyzed with proportion and frequency. Pearson chi‐square test or Fisher Exact (observation <5) was used to assess the relationship between receptor status and metastasis to the chest, abdominopelvic, bone, and LNs. Fisher exact test was used to determine whether LN participation in the cancer's spread to the chest and abdominopelvic region was substantial. The results are presented in tables and charts.

## 3 RESULTS

### 3.1 Characteristics of patients with advanced BC

The median age (interquartile range) of the patients with metastasis to the chest region was 46 (41–55) years, and the majority (55.6%) were 45 years old and above. Most patients were diagnosed with invasive carcinoma of no special type BC (97.2%). A higher proportion of patients had histological grade 3 (41.7%) and pathological stage IV (83.3%) tumors. The majority of breast tumor subtypes were characterized by negative receptor status: HER 2− (72.2%), ER− (55.6%), and PR− (69.4%). The triple‐negative (HER 2−/ER−/PR−) subtype was observed in 55.6% of the patients (Table 1).

**TABLE 1:** Demography and pathological characteristics of patients.

Variable	Number (*n*)	Percentage (%)
Age group		
Median (IQR)	46 (41–55)	
<45 years	16	44.4
≥45 years+	20	55.6
Breast tumor type		
Invasive ductal BC	35	97.2
Metaplastic BC	1	2.8
Laterality of primary tumor		
Left	21	58.3
Right	15	41.7
Pathological grade of breast cancer		
Grade 1	7	19.4
Grade 2	14	38.9
Grade 3	15	41.7
Pathological stage of breast cancer		
IIIB	7	16.7
IV	29	83.3
HER 2		
HER negative	26	72.2
HER positive	10	27.8
ER		
ER negative	20	55.6
ER positive	16	44.4
PR		
PR negative	25	69.4
PR positive	11	30.6
Triple negative		
No	20	55.6
Yes	16	44.4

Abbreviations: BC, breast cancer; ER, estrogen receptor; HER 2, human epidermal growth factor receptor 2; IQR, interquartile range; PR, progesterone receptor.

### 3.2 Summary of advanced BC radiological investigations

**FIGURE 1:**
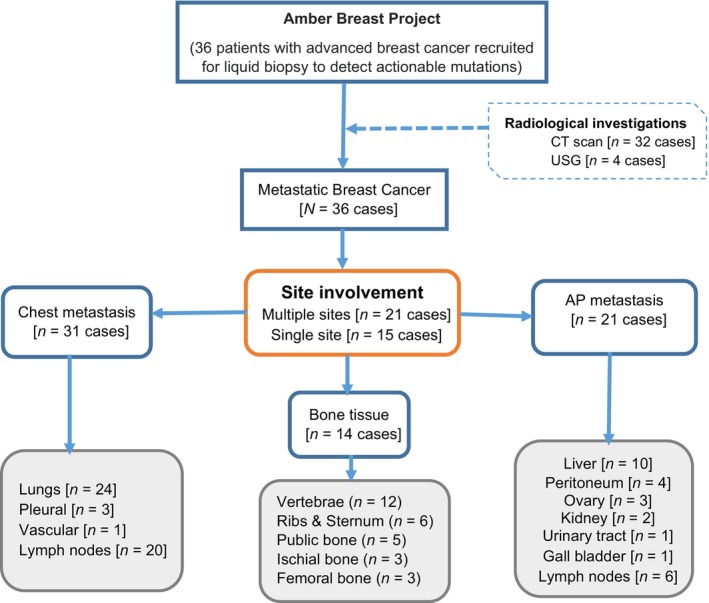
Flowchart showing the metastatic pattern on imaging among patients with advanced breast cancer. AP, abdominal and pelvic; CT, computed tomography; USG, ultrasonography.

Of the 36 patients, 31 (86.1%), 21 (58.3%), and 14 (38.8%) were diagnosed with chest, AP, and bone metastases, respectively. LN involvement was reported in 26 patients (20 in the chest and 6 in the AP) (Figure 1).

**FIGURE 2:**
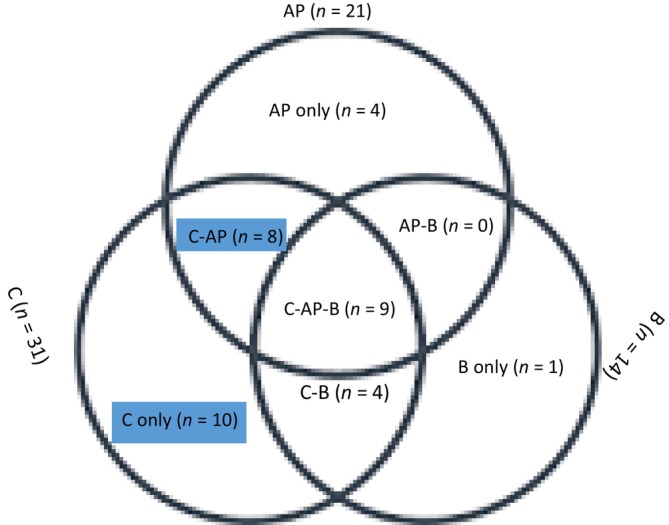
A Venn diagram to show multiple metastasis. AP, abdomen pelvis; B, bone; C, chest.

The majority, 21 (58.3%) had multiple sites of metastasis, with 15 (41.7%) showing single‐site metastasis. Triple‐site metastasis (C‐AP‐B) was observed in nine (24.3%) patients. Multiple‐site metastases involving the chest bones and chest–abdominopelvic lesions were observed in 13 (35.1%) and 17 (45.9%) patients, respectively (Figure 2).

### 3.3 Chest metastasis

**FIGURE 3:**
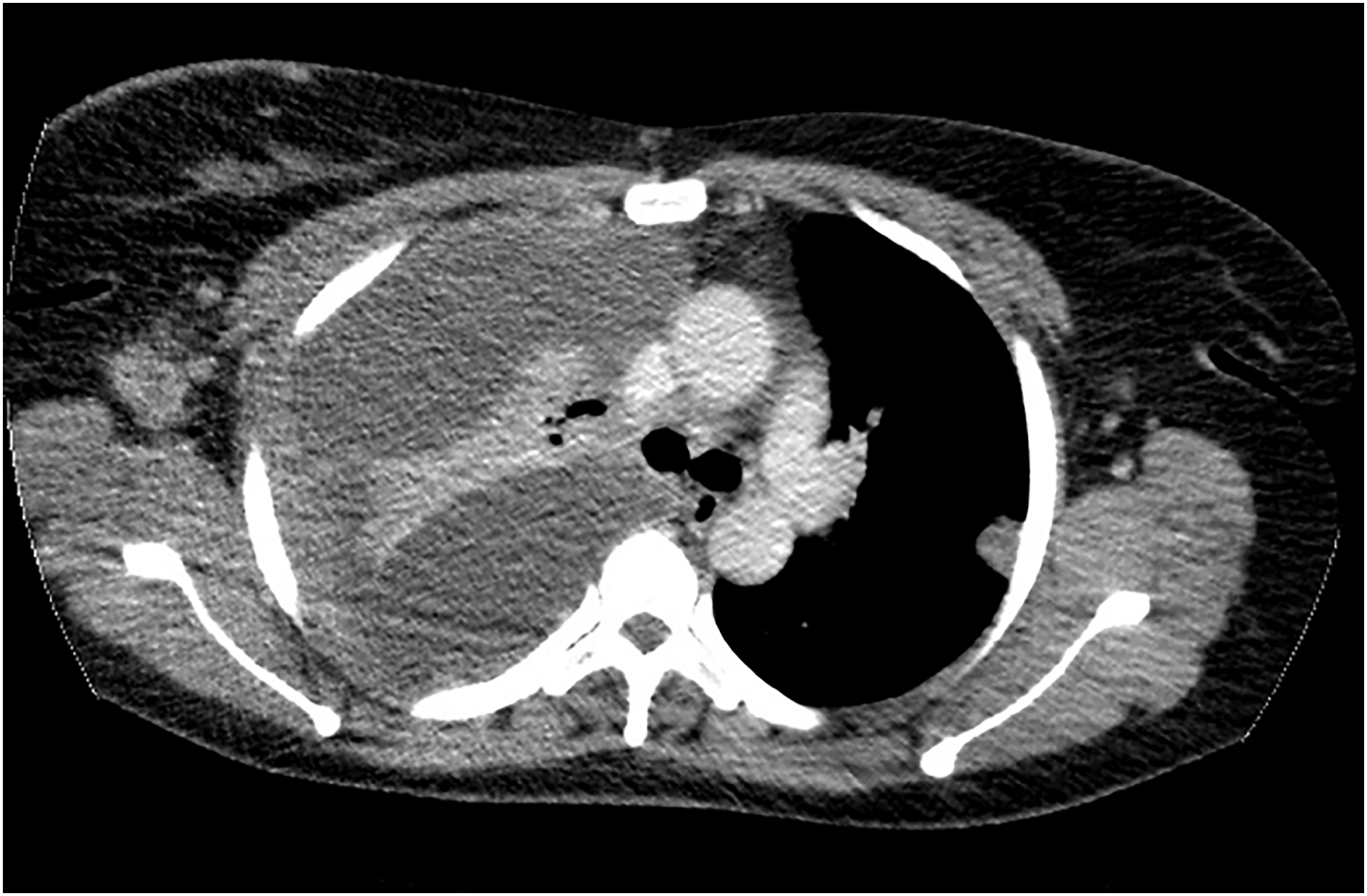
Axial computed tomography scan image of the chest, mediastinal window showing massive right pleural effusion, pleural thickening and left pleural nodule, highly suggestive of metastatic disease.

Of the 31 patients diagnosed with chest metastasis, the lungs (24, 77.4%) and lymph nodes (20, 64.5%) were the most frequent sites of metastasis. Only three (9.7%) and one (3.2%) patient showed pleural effusion and pulmonary artery metastasis, respectively. Figure 3 shows an axial CT scan image of the chest, mediastinal window showing massive right pleural effusion, pleural thickening, and left pleural nodule, highly suggestive of metastatic disease.

Most of the lung metastases were nodular (22, 91.7%), while 4 (16.7%) showed consolidation. Of the 22 patients with nodular lesions, 18 (81.8%) and 4 (18.2%) had single and multiple nodules, respectively. The upper lobes (bilateral: 45.5% [10/22], right: 40.9% [9/22], and left: 9.1% [2/22]) were the most common locations for nodules in patients with lung metastasis (Table 2).

**TABLE 2:** Morphology of lungs metastasis.

Features	Number (*n*)	Percentage (%)
Morphology of lung lesions (*n* = 24)		
Nodules	22	91.7
Consolidation	4	16.7
Lymphangitic	0	0.0
Endobronchial	0	0.0
Single nodules^a^ (*n* = 22)		
Yes	4	18.2
No	18	81.8
Multiple nodules^a^ (*n* = 22)		
Yes	18	81.8
No	4	18.2
Location nodules (*n* = 22)		
Right upper lobe	9	40.9
Right lower lobe	3	13.6
Left upper lobe	2	9.1
Left lower lobe	3	13.6
Bilateral upper lobe	10	45.5
Bilateral lower lobe	7	31.8
Middle lobe	2	9.1

^a^ A patient could present with single or multiple nodules in different lobes.

### 3.4 Abdominopelvic metastasis

**FIGURE 4:**
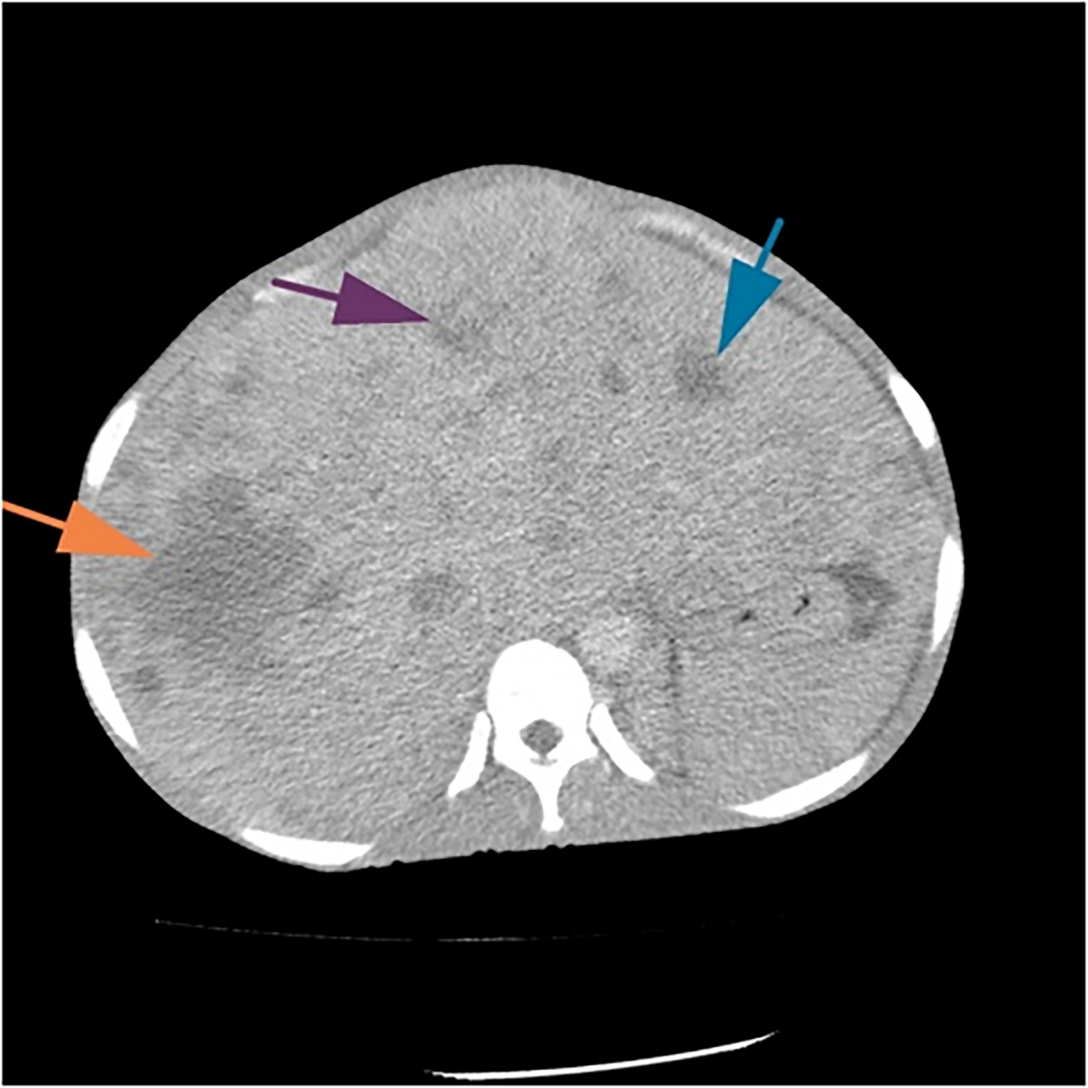
Axial computed tomography images of the abdomen, at the level of the liver. The image shows innumerable hypodense minimally enhancing lesions (arrows), consistent with metastasis.

Of the 21 patients with AP metastasis, 18 (85.7%) were presented with metastases to multiple organs. Figure 4 shows the axial CT images of the abdomen at the level of the liver. The image shows numerous hypodense, minimally enhancing lesions (arrows), consistent with metastasis. Liver metastasis (*n* = 10, 47.6%) was the most frequently observed lesions. Other metastatic sites were the peritoneum (*n* = 4, 19.1%) and the ovary (*n* = 2, 9.5%).

The radiological features of liver metastases are shown in Table 3. Of the 10 patients with liver metastasis, a hypodense mass was seen in four cases, a heterogeneous mass in five cases and a hypoechoic mass in one case. Regarding the distribution of metastatic deposits, four cases each had diffuse and focally distributed masses. A heterogeneous enhancing mass was seen in seven cases while a homogeneously enhancing mass was seen in three cases. Seven patients had an enlarged liver; necrotic and pleomorphic masses were seen in three (Table 3).

**TABLE 3:** Radiological features of liver metastasis in advance breast cancer patient.

Feature of mass	No. of ABC patients (*n* = 10)	Percentage (%)
Morphology of lesions		
Hypodense	4	40.0
Heterogeneous	5	50.0
Hypoechoic	1	10.0
Distribution of lesions		
Diffuse	5	50.0
Focal	5	50.0
Margins of lesions		
Ill‐defined	2	20.0
Well‐defined	6	60.0
Mixed margin	2	20.0
Enhancement		
Heterogeneous	7	70.0
Homogeneous	3	30.0
Necrosis		
No	7	70.0
Yes	3	30.0
Enlarged liver		
No	3	30.0
Yes	7	70.0
Pleomorphism		
No	7	70.0
Yes	3	30.0
Predominant shape		
Indeterminate	6	60.0
Round	4	40.0

Abbreviation: ABC, advance breast cancer.

### 3.5 Bone metastases

**FIGURE 5:**
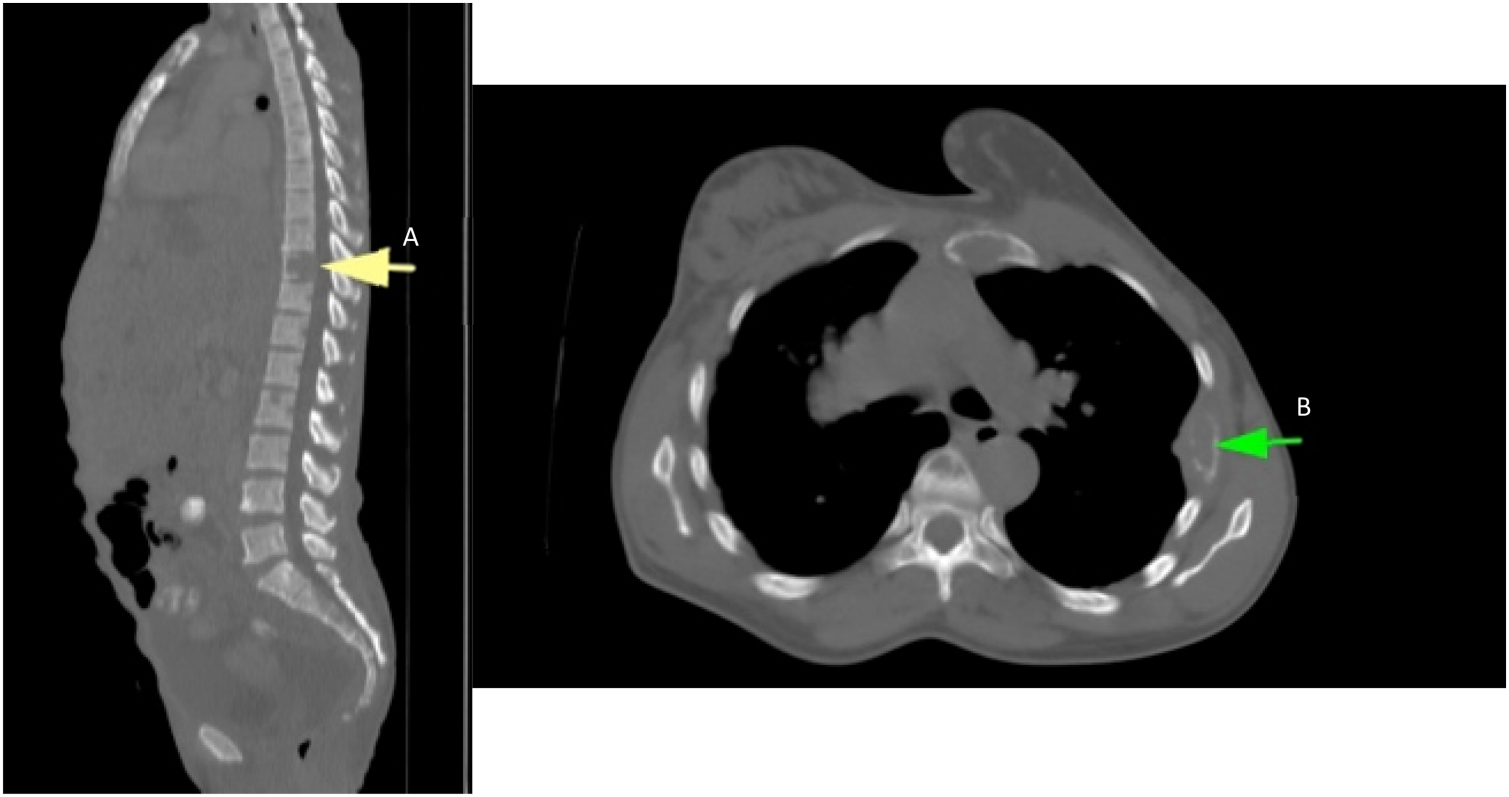
Sagittal (A) and axial (B) computed tomography scan images of the spine and chest, bone windows, showing lytic lesions in body of T10 vertebra and left rib (arrows).

The radiographic features of all 14 patients (37.8%) with bone metastasis were assessed. Of the 14 bone metastasis cases, 13 (92.9%) were lytic lesions and only one (7.1%) sclerotic lesion was observed. One patient had metastatic soft tissue deposits. Figure 5 shows the sagittal (A) and axial (B) CT scan images of the spine, chest, and bone windows, showing lytic lesions in the body of the T10 vertebra and left rib.

Narrow, wide, and mixed zones transitions were reported in 9 (64.3%), 3 (21.4%), and 2 (14.3%) cases, respectively. The margins of the lesions were examined in 14 cases: six (42.9%) had well‐defined lesions, four (28.6%) had lesions with ill‐defined margins, and four (28.6%) had mixed margins. The majority of cases were diagnosed as multiple bone lesions (*n* = 12, 85.7%). Bone metastases were common to the vertebrae (thoracic [*n* = 11, 78.6%], lumbar [*n* = 7, 50.0%], sacral [*n* = 6, 42.9%], and cervical [*n* = 2, 14.3%]). (Table 4).

**TABLE 4:** Radiographic appearance of bone metastases.

Features	Frequency (*n* = 14)	Percentage (%)
Type of lesion		
Lytic	13	92.9
Sclerotic	1	7.1
Zone of transition		
Narrow	9	64.3
Wide	3	21.4
Both	2	14.3
Margins		
Ill‐defined	4	28.6
Well‐defined	6	42.9
Both	4	28.6
Number of lesions		
Single	2	14.3
Multiple	12	85.7
Affected bones		
Thoracic vertebrae	11	78.6
Lumbar vertebrae	7	50.0
Iliac bone	7	50.0
Sacral vertebrae	6	42.9
Public bone	5	35.7
Ischial bone	3	21.4
Femoral bone	3	21.4
Ribs	3	21.4
Sternum	3	21.4
Cervical vertebrae	2	14.3
Clavicle	1	7.1
Acetabulum	1	7.1

*Note*: Only one patient showed soft tissue involvement.

### 3.6 Lymph node metastasis

**FIGURE 6:**
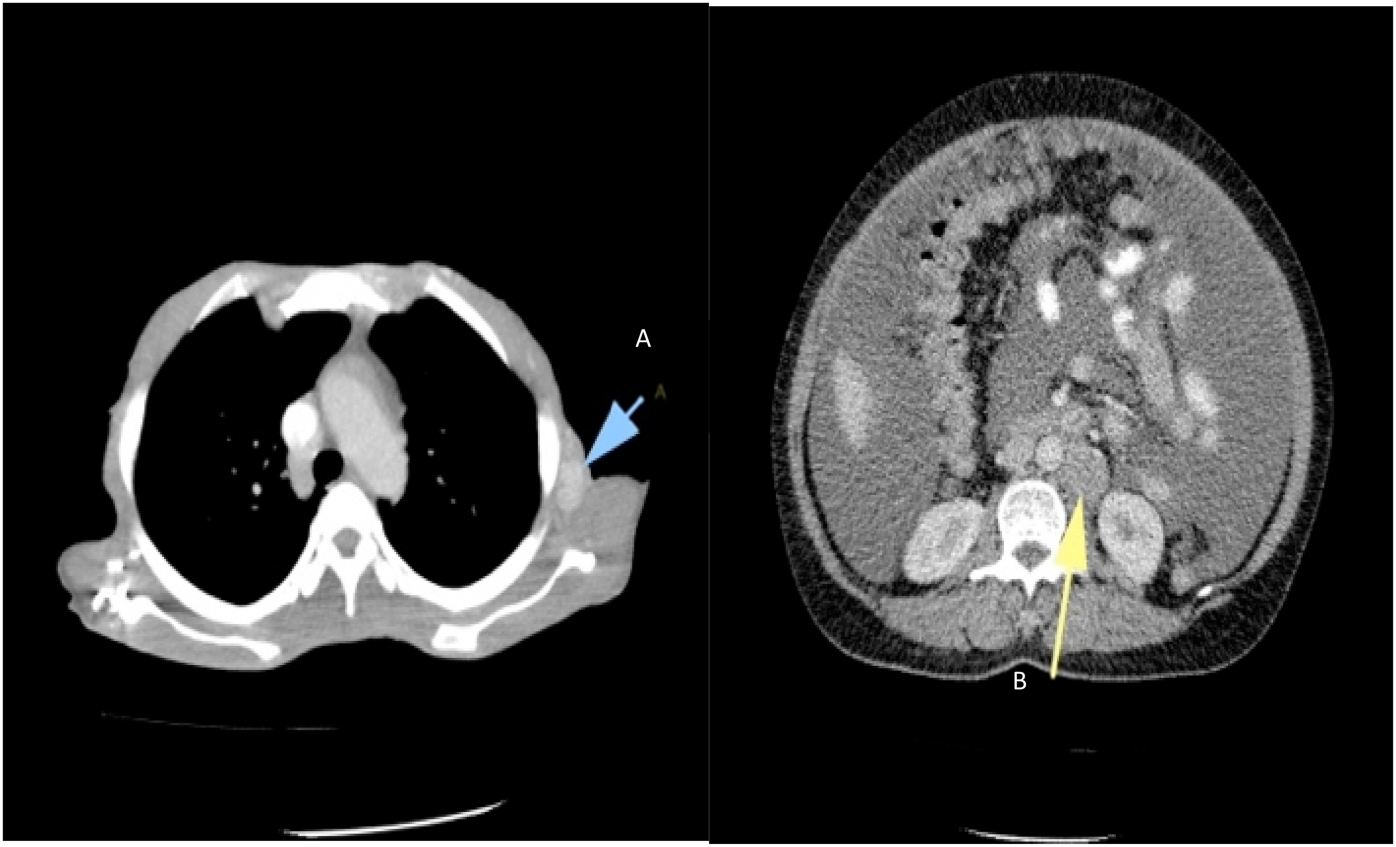
Axial computed tomography scan of the abdomen and chest shows enlarged round/oval lymph nodes in the axilla (A) and portal hepatis (B).

Metastatic LNs were more common in the chest (20, 54.1%) than in the AP (6, 16.2%). Multiple LNs were seen in seven (19.1%). Figure 6 shows an axial CT scan of the abdomen and chest showing enlarged round/oval lymph nodes in the axilla (A) and portal hepatis (B).

**FIGURE 7:**
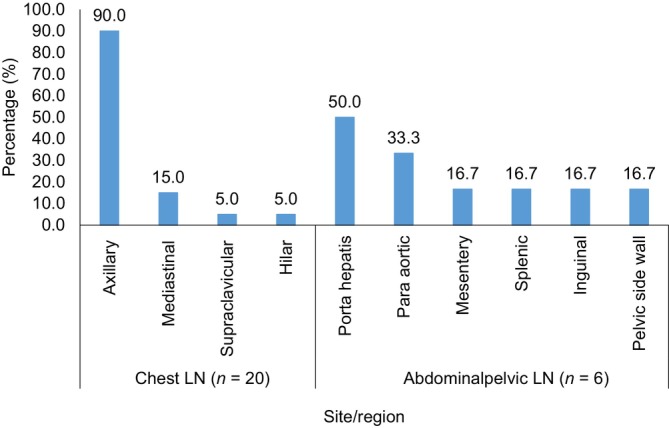
Sites of metastatic lymph nodes (LNs) in advance breast cancer patient.

Axillary LN (18, 90.0%) and porta hepatis LN (3, 50.0%) were the most common LN metastases detected in the chest and AP, respectively (Figure 7). The LN were predominantly single and well defined, with mild to moderate postcontrast enhancement. A test of association to determine whether LN participation in the cancer's spread to the chest and abdominopelvic region was substantial. LN involvement was significantly correlated with chest metastasis (*p* = .012), but not with abdominopelvic metastasis (*p* = .065).

### 3.7 Correlates of type of metastatic

Metastasis involving multiple sites (12, 57.1%), bone (8, 57.1%), and abdominopelvic (12, 57.1%) was high among patients aged <45 years. There was no significant association between age and the type of metastatic tumor (Table 5).

**TABLE 5:** Association between age and type of metastasis.

Variable	<45 years	≥45 years	*p*‐value
Number of sites involved			.096^#^
Single	4 (26.7)	11 (73.3)	
Multiple	12 (57.1)	9 (42.9)	
Chest metastasis			.355^#^
No	1 (20.0)	4 (80.0)	
Yes	15 (48.4)	16 (51.6)	
Bone metastasis			.221^a^
No	8 (36.4)	14 (63.6)	
Yes	8 (57.1)	6 (42.9)	
Abdominopelvic metastasis			.096^#^
No	4 (26.7)	11 (73.3)	
Yes	12 (57.1)	9 (42.9)	

^#^ Fisher's exact test *p*‐value.

^a^ Chi‐square test.

Grade 3 tumors were common among the patients with multiple sites (10, 47.6%), chest (13, 41.9%), bone (6, 42.9%), and abdominopelvic (11, 52.4%) involvement. No significant association was found between the grade of BC according to histology and type of metastasis (Table 6).

**TABLE 6:** Association between grade of breast cancer by histology and type of metastasis.

Type of metastasis	Grade 1	Grade 2	Grade 3	*p*‐value
Number of sites involved				.316^#^
Single	2 (13.3)	8 (53.3)	5 (33.3)	
Multiple	5 (23.8)	6 (28.6)	10 (47.6)	
Chest metastasis				.407^#^
No	0 (0.0)	3 (60.0)	2 (40.0)	
Yes	7 (22.6)	11 (35.5)	13 (41.9)	
Bone metastasis				.446^#^
No	3 (13.6)	10 (45.5)	9 (40.9)	
Yes	4 (28.6)	4 (28.6)	6 (42.9)	
Abdominopelvic metastasis				.250^#^
No	3 (20.0)	8 (53.3)	4 (26.7)	
Yes	4 (19.0)	6 (28.6)	11 (52.4)	

^#^ Fisher exact test *p‐*value.

## 4 DISCUSSION

BC metastases of the distant organs negatively influence treatment outcomes.^6^, ^15^ To assess metastasis, the thoracic organs, soft tissues in the abdomen and pelvis, and associated bones could all be examined with CT scans.^6^ Previous studies report that metastatic BC is present in 34% of women with BC seen in Ghanaian tertiary health facilities.^15^ The lung, bone, and liver, are the most common sites of metastasis for BC.^6^, ^25^ The reported incidence is higher in the chest region than AP.

Research has confirmed the impact of geopolitical factors such as socioeconomic dynamics of the populace, on the utilization of CT scans by BC patients. Principal among these factors was financial constraints such as the cost of a CT scan and distance as major barriers to BC patients receiving CT scans.^26^, ^27^, ^28^ However, in the current study, one CT scan per participant was required, any direct costs related to CT scan acquisition were sponsored to remove financial barriers. Hence, we believe our intervention of sponsoring patients' costs of CT scans and transportation to and from the CT scan center has controlled the effect on the prevalence of metastases in a patient.

The prevalence of chest metastasis was higher among patients with BC in Ghana; however, there is little to no evidence on its radiological incidence and characteristics. Our study has revealed that 32 (86.5%) of the patients reviewed had metastasis to the lungs, axillary LN, pleural, and pulmonary artery. This is consistent with previous reports in metastatic BC.^29^, ^30^

In previous reports, lung metastasis was the second most frequent distant metastasis in BC and the general population.^3^, ^29^ However, in this study, lung metastasis was the foremost distant metastasis with more than two‐thirds (66.7%) of our patients diagnosed with lungs metastasis. The rate of lung metastasis is comparable to 36%–80% of that in previous studies.^29^, ^30^, ^31^ Lung metastases are notably high in women aged 50 years and above, with a reported incidence above 80%.^30^ The high incidence of lungs metastasis has been linked to age at BC cancer diagnosis, advanced stage, bone metastasis, and tumor receptor status such as HR+/HER2− and triple negative.^29^ The present study found no significant association between chest or lungs metastasis and the patient age, tumor grade, or receptor status. A review of patients' clinical history also showed that only seven of these patients had a medical history of hypertension, which had no significant relationship with the presence of lung metastasis. However, Kocher et al. however reported an association between hypertension and hyperlipidemia and the likelihood of lung metastasis in BC patients using artificial intelligence as a prognostic tool for precision medicine.^26^

Different radiological features have been documented.^23^, ^32^, ^33^, ^34^ In most of our cases, the imaging assessment of lung lesions was reported as nodular, with only four cases presenting as consolidation. No lymphangitic or endobronchial lesions were observed. There was a series of multiple nodular masses in 18 of these patients, which were commonly found in the upper lobes of the lung. The most common type is solitary pulmonary nodule, multicentric or diffuse disease, and a localized area of parenchymal consolidation.^23^

In this study, 59.5% of the patients were diagnosed with AP metastasis, and the liver, peritoneum, and ovaries were the most affected organs. These findings are comparable to the reported patterns of AP metastasis by Patnaik et al.,^12^ who reported 52.3% in their cohort at their first presentation, with the liver, mesentery, and peritoneum/omentum identified as the most affected sites.^12^

A previous study reported that 71% of BC patients who died had metastases in the liver.^7^ However, the proportion attributed to BCs is generally not known. A study in India reported liver metastasis in 28% of 42 BC cases.^12^ Liver involvement in the current study was 45.5% among patients with AP metastasis. The prevalence of diffuse and focally distributed liver masses was comparable to the report of diffuse infiltration of the liver with metastatic disease by Brookes et al.^7^ Patients with more locally advanced BC should have investigations focused on finding underlying asymptomatic metastases, as this may influence management.^35^

The two most common liver metastatic lesions were hypodense and heterogeneous masses. Six of the liver masses had well‐defined margins, two had ill‐defined margins, and two had mixed margins. Typical features of metastases in the liver are an irregular, ill‐defined, low‐attenuation area within the liver parenchyma that is best imaged during the portal venous phase.^7^ An enhancing liver mass is a common finding in the CT diagnosis of metastatic tumors.^12^ In this study, a heterogeneous enhancing mass was observed in seven cases, whereas three cases had homogeneous enhancing masses. The diagnosis of lesion enhancement could suggest the disease's progression.^5^, ^12^ Enlarged liver, necrotic liver mass, and mass pleomorphism were the other radiologic patterns of liver metastasis. While an enlarged liver is a common finding on liver imaging, necrotic liver masses and pleomorphic masses are unusual.

Bone is the most common site for BC metastasis.^20^ Isolated bone metastasis is rare. Most of the time, it is associated with liver and peritoneal spread.^12^ Nearly 38% of our patients had bone metastasis. Despite this high incidence, it is reported that over 80% of BC patients are likely to be diagnosed with bone metastases in postmortem studies across different cancers.^36^

Locally, late stage (3 and 4) patients receive chemotherapy and antiestrogen therapy along with radiation therapy to improve health and enhance quality of life (QoL).^15^, ^37^, ^38^, ^39^ A study among a cohort of 184 patients by Okifo et al. in the same facility revealed that Stage 3 and 4 patients were two‐fold more likely to receive neoadjuvant chemotherapy than earlier stages. But we found no statistical association between other chemotherapies, radiation therapy, oral antiestrogen medication administration, or stage of the disease. However, studies did not assess the level to which available treatments at the facility improve the health and QoL of their patients. Meanwhile, a study of the change in QoL of metastatic BC patients seeking treatment at Komfo Anokye Teaching Hospital by Agbeko et al. indicated some improvement in mortality rates and QoL among patients who received varying treatment courses as compared to baseline data.^38^ Also, Baako and Badoe found a significant improvement in the survival rate of patients with advanced BC after chemotherapy postsurgical intervention.^39^

Cook, et al. noted that bones are frequent sites of spread in advanced BC.^21^ This could lead to high morbidity and unbearable health care costs, especially in resource‐limited settings like Ghana.^21^ Despite the cost imposed by additional CT scan, there are sufficient data to support the value of a CT scan in determining whether BC has spread to distant organs in the chest, abdomen, and pelvis.^6^, ^24^, ^40^, ^41^ It is noted that when evaluating metastases in the chest, abdomen, and pelvic regions, bone involvement discovered by CT scan was an accidental finding. This emphasizes the value of CT scan above normal palpation and mammography for localized breast tumors. In our cases with advanced metastatic BCs involving distant organs requiring other methods of assessment such as CT scan, Ultrasonography (US), Magnetic Resonance Imaging (MRI), and so forth, the assertion that “previous study has reported that adding additional modalities other than palpation and mammography to BC follow‐up does not improve prognosis” is inconsistent with the current study.^42^ Relatively inexpensive imaging is required to evaluate the characteristics of metastasis in cases of advanced metastatic cancer, in which the original tumor has progressed to distant organs in the chest, abdomen, and pelvic regions. Features that are not apparent with palpation or mammography, such as shape, size, number, sclerotic/lytic, soft tissues association, and presence and nature of lung nodules, are enhanced by a CT scan. The advantage of using a CT scan to detect metastatic tumors is that it allows for more targeted treatment to improve a BC patient's health and QoL.^9^, ^43^, ^44^

The majority of the cases had multiple bone lesions, with only two having one, which corroborates previous findings that bone metastases most commonly involve the vertebrae and pelvis^12^, ^36^ Most of the bone metastases seen involved the thoracic, lumbar, sacral, and cervical vertebrae are predominantly lytic. The vertebrae contain the red marrow in the adult that could promote metastasis.^36^ The properties of the circulation, cells, and extracellular matrix within this region could assist in the formation of bone metastases.^32^ The frequent metastasis of the axial skeleton and limb girdles in BC may be partially attributed to the flow of blood to the skeleton through the vertebral‐venous plexus.^20^ Though our study showed 38% of bone metastasis, most were incidental findings from the abdominal and chest CT scans. The use of CT scans in detecting bone metastasis has low sensitivity and specificity rates.^45^ More lesions may have been detected using other imaging modalities such as MRI and PET scans.^45^

LNs play a crucial role in the control of tumor progression, as they present the best opportunity for primary tumor diagnosis via histologic and imaging evaluation.^46^ Therefore, careful assessment of LN metastases is the key when staging carcinoma of the breast.^7^ A significant proportion of our BC patients had multiple LN metastasis. This suggests multiple lymph drainage in patients with BC. Consistent with existing studies,^2^, ^47^ the majority of our study sample with chest metastasis involved the axillary LN. Axillary nodes remain the most preferable lymph drainage of the breast underscoring the high incidence of axillary LN metastasis in BC patients.^35^, ^46^, ^48^ This might have explained why there was a significant correlation in the LN involvement with chest metastasis. Although the preferential lymph drainage of the breast is to the axillary nodes, nodal involvement at distant sites, including the AP, may occur in more local or intra‐abdominal nodes.^6^, ^7^ In this study, abdominal LN metastasis occurred in five patients, representing 22.7% of the 22 cases with AP metastasis. This was much higher than the reported prevalence of LN metastasis, which ranges from 1.1% to 3.4% in patients with AP tumors.^12^

Evidence from previous studies may suggest that liver and pancreatic metastasis are major causes of the spread of invasive tumor cells to LNs in the AP region.^12^, ^49^ Secondary drainage for most AP tumors occur via the porta hepatis, common hepatic, coeliac, and mesenteric root LNs.^12^, ^46^ The vascular mechanism and lymphatic involvement therefore encourage the spread of invasive cancer cells to the lymphatic drainage system.^46^, ^47^, ^49^

The key limitations noted were the sample size, number of subjects, and the single facility involved. The study involved only patients with advanced BC from one teaching hospital in Ghana and was therefore not representative of the Ghanaian population. Hence, the findings may not be generalizable to other centers in Ghana, as patients served by other tertiary facilities may present a different pattern of metastasis due to sociodemographic characteristics, CT scan quality, and the accuracy of imaging interpretations. The study is only a metastasis positivity rate based on imaging criteria determined by the authors based on previous studies.

## 5 CONCLUSION

Advanced BC may present with multiple metastases. There was a high radiologic incidence of BC metastasis to the lungs, liver, bone, and lymph nodes in our late‐stage BC cohort. There was no statistically significant relationship between the tumor characteristics and the likelihood of metastasis to a specific distant site. Additional studies with a larger number of patients may establish existing relationships and allow for more appropriate patient management, in the case where MRI is the imaging modality of choice for HER‐2 positive patients because metastatic brain tumors are more common in these patients.

## AUTHOR CONTRIBUTIONS

**Bashiru Babatunde Jimah:** Conceptualization (equal); data curation (equal); formal analysis (lead); methodology (equal); resources (equal); writing – original draft (lead); writing – review and editing (equal). **Emmanuella Amoako:** Conceptualization (lead); data curation (equal); investigation (equal); project administration (lead); supervision (lead); writing – review and editing (equal). **Emmanuel Owusu Ofori:** Data curation (equal); writing – review and editing (equal). **Patrick Kafui Akakpo:** Conceptualization (equal); writing – review and editing (equal). **Luke Adagrah Aniakwo:** Data curation (equal). **Kofi Ulzen‐Appiah:** Data curation (equal). **Emmanuel Gustav Imbeah:** Data curation (equal). **Martin Tangnaa Morna:** Conceptualization (equal). **Patience Koggoh:** Data curation (equal). **Harry Akligoh:** Data curation (equal). **Randy Tackie:** Data curation (equal). **Aida Manu:** Conceptualization (equal); data curation (equal); writing – review and editing (equal). **Lily Paemka:** Conceptualization (equal); writing – review and editing (equal). **Benjamin Dabo Sarkodie:** Data curation (equal); writing – review and editing (equal). **Asare Kweku Offei:** Data curation (equal); writing – review and editing (equal). **David Hutchful:** Data curation (equal); writing – review and editing (equal). **Joyce Ngoi:** Conceptualization (equal); writing – review and editing (equal). **Yaw Bediako:** Conceptualization (lead); data curation (equal); funding acquisition (lead); project administration (lead); resources (lead); writing – review and editing (equal). **Ganiyu Adebisi Rahman:** Conceptualization (equal); methodology (equal); writing – review and editing (equal).

## FUNDING INFORMATION

The study was funded by Yemaachi Biotech and radiological support from RAAJ Diagnostics. Author Yaw Bediako is the CEO of Yemaachi Biotech. Author Bashiru Babatunde Jimah is the CEO of RAAJ.

## CONFLICT OF INTEREST STATEMENT

The authors have stated explicitly that there are no conflicts of interest in connection with this article.

## ETHICS STATEMENT

This study is part of the AMBER01 study, which was approved by the ethical review committee (CCTHERC/EC/2021/051). Data were maintained in a password‐protected database stored on an encrypted server (UVOSYO) with access to the investigators only.

## Data Availability

The data used to support the findings of this study are included within the article.
